# CURRENT CONCEPTS IN THE PATHOGENESIS OF PSORIASIS

**DOI:** 10.4103/0019-5154.48977

**Published:** 2009

**Authors:** Rajeev Patrick Das, Arun Kumar Jain, V Ramesh

**Affiliations:** *From the Institute of Pathology (ICMR), Safdarjang Hospital and Vardhman Mahavir Medical College, New Delhi, India*; 1 *From the Department of Dermatology and Regional STD Centre, Safdarjang Hospital and Vardhman Mahavir Medical College, New Delhi, India*

**Keywords:** *Autoimmunity*, *pathogenesis*, *psoriasis*

## Abstract

Psoriasis is a multi-factorial skin disease with a complex pathogenesis. Various factors which have been suggested to play a key role in the pathogenesis are T cells, antigen presenting cells (APC's), keratinocytes, Langerhans' cells, macrophages, natural killer cells, an array of Th1 type cytokines, certain growth factors like vascular endothelial growth factor (VEGF), keratinocyte growth factor (KGF), and others. It has been hypothesized that the disease starts with the activation of T cell by an unknown antigen, which leads to secretion of an array of cytokines by activated T cells, inflammatory cells, and keratinocytes. The characteristic lesion of psoriasis is due to the hyper-proliferation of the keratinocyte. Activated Langerhans' cells migrate from skin to lymph nodes presenting the antigen to nodal naïve T cells (cells that have not been activated by antigen previously). The T cells activated by non-antigen-dependent mechanism may, however, become antigen-specific memory cells that react with a cross-reactive auto-antigen such as keratin (molecular mimicry). The genetic background of the disease may be suggested from the fact that concordance rate is 63–73% in monozygotic twins, as compared to 17–20% in dizygotic twins. Several disease susceptibility loci have been suggested as predisposing factors, PSORS1-PSORS9.

## Introduction

Whether psoriasis represents a fundamental disease of skin, or the immune system, has been debated for several years. Role of T cells, antigen-presenting cells, keratinocytes, Langerhans' cell, macrophages, natural killer cells, an array of Th1 type cytokines, certain growth factors like vascular endothelial growth factor (VEGF) keratinocyte growth factor (KGF) and others have been suggested to play a key role in pathogenesis of psoriasis. Currently a wide array of treatment modalities is available for psoriasis, but with every treatment there is a possibility of remission. Systemic or UV photo-therapies may have unacceptable side effects; hepatotoxicity and nephrotoxicity may follow treatment with methotrexate or cyclosporine; teratogenicity is a risk of oral retinoids, and skin cancer may be caused by frequent PUVA (Psoralen and long wave ultraviolet radiation) treatments.[1,2] In the past decade researchers have come up with new factors that may be involved in the pathogenesis of disease, but they have failed to establish a pathogenetic model that incorporates all the factors. There is a need to review our current concept and understanding of the pathogenesis of psoriasis. Lack of a clear pathogenetic mechanism especially in remissions, makes it very difficult to manage the disease on long term. This article is an attempt to bridge the gap that has come up with recent studies and to establish a better understanding of the pathogenesis of disease.

## T Cell Activation

**Figure 1:**
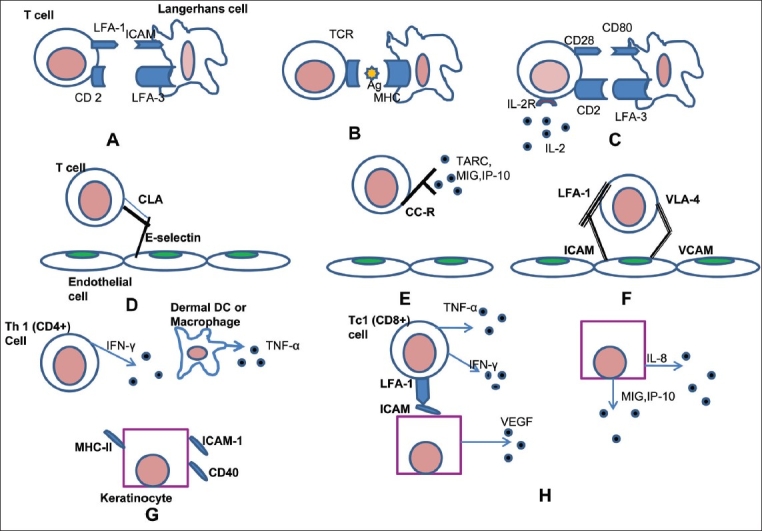
The current hypothesis by which T cells get activated and how the release of various mediators leads to the hyperproliferation of the keratinocyte. (Modified from Mehlis and Gordon^1^, Krueger^37^) (A) T cell binds to an Antigen-Presenting Cell, (B) T cell receptor recognizes the antigen presented on MHC of the APC in an antigen specific interaction, (C) Non-antigen specific cell-interaction. The stimulation of both TCR and CD28 pathways lead to transcription of IL-2, TNF-α, GM-CSF and IFN-Y, (D) T cell is rolling on the endothelium, (E) T cell surface proteins are activated, (F) T cell binds to the endothelium and diapedesis occurs, (G) Dermal Th1 cells release IFN-Y and other cytokines, which lead to increases expression of inflammatory and adhesion proteins on keratinocytes, (H) Keratinocytes proliferate; synthesize angiogenic cytokines / chemokines that cause leukocyte trafficking and increase leukocyte adhesion to the endothelial cells.

It is widely believed that abnormal regulation of T cells coupled with interaction between keratinocytes and complex cytokine network is involved in the pathogenesis of the disease.[3,4] In case the primary defect resides in keratinocytes, any physical or chemical injury to the defective keratinocytes could activate synthesis and release of cytokines thereby resulting in antigen-independent activation of T lymphocytes. This would further lead to release of additional cytokines followed by proliferation of keratinocytes, T lymphocytes and inflammation. Chang *et al*[5] have demonstrated that cytokines secreted by psoriatic epidermal cells potentiate T lymphocyte activation to a greater extent than cytokines secreted from normal epidermal cells. It is also postulated that only psoriatic keratinocytes respond to activated T cell messages with hyper-proliferation, because of their specific receptors or signal-transducing mechanisms [Figure 1].[4] Further normal keratinocytes do not respond to psoriatic T cell supernatants.[6]

According to some studies the basement membrane (BM) structures are altered, and a complex network of cytokines, mainly Th1 type, are involved in various stages of pathogenesis.[1–3] Several new treatments reducing or eliminating the pathogenic effects of T cells are being investigated as possible anti psoriatic drugs. Alefacept binds to CD2 on T cells, blocking the LFA-3/CD2 interaction.[7] Alefacept also binds to FcgRIII IgG receptors on natural killer (NK) cells and macrophages, resulting in apoptosis of those T cells expressing high levels of CD2. Efalizumab is an antibody directed against the alpha subunit of LFA-1.[3,4,7] Etanercept and infliximab act as competitive inhibitors of tumour necrosis factor-alpha (TNF-α), which is an important pro-inflammatory cytokine in the pathobiology of psoriasis.[8,9] Unfortunately, only one-third patients get significant benefit from the new and expensive immunomodulatory drugs. At least in the long term, increased risk of infection and possible reactivation of tuberculosis and lymphomas must be taken into account, as these new drugs are potentially immunosuppressive.[8]

## Hyperproliferation of Keratinocytes

The cell cycle time of hyperproliferating psoriatic keratinocytes is short. While maturation and shedding of keratinocytes takes 26 days in normal epidermis, it occurs in 4 days in psoriatic epidermis.[9] Growth factors, coming from various cell types, are believed to control the increased proliferation. Currently available antipsoriatic drugs act on keratinocyte proliferation. Calcipotriol, a vitamin D3 analog and retinoids, the natural and synthetic vitamin A derivatives, modulate keratinocyte hyperproliferation and differentiation.[10] Cyclosporine has strong antiproliferative effects on human epidermal keratinocytes in addition to immunomodulatory effects.[11]

## Angiogenesis

Keratinocytes are thought to be a major source of pro-angiogenic cytokines (VEGF, IL-8) but the precise mechanism for angiogenesis in psoriasis is still unknown. In a developing psoriatic plaque, endothelial cells swell and become activated showing prominent Golgi apparatus and Weibel-Palade bodies.[12] Activated endothelial cells migrate, sprout, and lay down a BM with pericytes for structural support to form novel vessel networks.[13] Activation and swelling of endothelial cells results in widening of the intercellular spaces, and dermal blood vessels dilate. The lesional capillary loops adopt a venous phenotype, including bridged fenestrations, and express E-selectin, making it easier for leukocytes to migrate into the skin.[14]

Although angiogenesis may not be the primary event in the pathogenesis of psoriasis, understanding the pathways leading to angio-proliferation may help in finding novel antipsoriatic drugs.[15] In fact, vitamin D analogues, retinoids, and cyclosporine all possess anti-angiogenic activity as well as antiproliferative and anti-inflammatory effects.[14,16]

We could also accredit the impact of some environmental factors on the induction of psoriasis symptoms. Despite the clear familial aggregation of psoriasis, the precise inheritance model has been under debate. Currently, most investigators agree that psoriasis belongs to the group of complex diseases, the inheritance being multifactorial – genetic variants in multiple genes interact both with each other and the environment.[17–20] Several disease susceptibility loci have been suggested as predisposing factors[21] [Table 1].

**Table 1:** Psoriasis susceptibility loci by genome-wide linkage scans

Locus ^[Reference]^	Location
PSORS1 [21–27]	6p21.3
PSORS2 [22,24,26,28]	17q24-q25
PSORS3 [29]	4q34
PSORS4 [30,31]	1q21
PSORS5 [26,32]	3q21
PSORS6 [33]	19p13
PSORS7 [32]	1p35-p34
PSORS8 [22,34]	16q12-q13

## Cytokine Mediators

Although a complex and multi-dimensional network of several cytokines has been found to be involved in pathobiology of psoriasis, none of these alone can be considered to be the causative.[35] Table 2 shows some of the key cytokines involved in the pathogenesis of psoriasis.

**Table 2:** Cytokines in the pathogenesis of psoriasis

Cytokine/Growth factor ^reference^	Role in psoriasis
TNF-α^8^,^9^,^35^,^36^	Stimulation of keratinocytes to produce IL-8, ICAM-1, TGF-α, β-defensins, GM-CSF and PAI2. Enhancement of pro-inflammatory cytokine secreting capacity of Macrophage. Stimulation of endothelial cell to secrete VEGF. Increased keratinocyte proliferation.
IFN-γ^35^,^37^,^38^	Antiproliferative effect on normal keratinocyte *in-vitro.* Induction of ICAM-1 expression on keratinocytes and endothelial cells, influencing the trafficking lymphocytes into lesional epidermis. Stimulation of APC activity and TNF-α release by phagocytes and up regulation of TNF-α receptors

GM-CSF^35^,^39^,^40^	Increases keratinocyte proliferation and activates neutrophils. It also stimulates migration and proliferation of endothelial cells.
IL-1^35^,^37^,^39^,^41^	Induction of E-selectin, VCAM-1, ICAM-1 on keratinocytes and expression of KGF and GM-CSF in fibroblasts. These fibroblast-derived factors in turn stimulate keratinocyte proliferation and differentiation. A direct keratinocyte mitogen, which mediates angiogenesis,
IL-2^35^–^42^	Is a growth factor and chemo-attractant for T cells Induces T cell cytotoxicity. Stimulates NK cell activity. High doses of IL-2 may induce psoriasis in predisposed patients.
IL-6^35^–^37^,^43^	Enhances the activation, proliferation, and chemotaxis of T lymphocytes in dermal infiltrate. Proliferation and activation of B cells and macrophages. Stimulation of keratinocyte proliferation *in vitro.*
IL-8^35^,^39^,^44^,^45^	Migration of neutrophils and T Cells in to epidermis Activation and proliferation of T lymphocytes and stimulation of angiogenesis.
IL-12^37^	Enhances T cell activation and differentiation stimulating the type 1 T cell maturation pathway.
EGF family^35^,^39^,^46^,^47^	Expression of TGF-α and amphiregulin is increased in psoriasis. Increased EGF/TGFα receptors in psoriatic epidermis. TGF-α induces IL-1, and has mitogenic and angiogenic properties.
VEGF^37^,^48^,^49^	Up-regulated in psoriasis causing erythema. Regulates vascular growth and remodelling in psoriasis lesions. Leukocytes show increased adhesion to selectins and VCAM expressed on new vessels in skin, and therefore VEGF may be the link between angiogenesis and cell-mediated inflammation in psoriasis.
FGF^4^,^14^,^50^	Has mitogenic and angiogenic properties and is found not only basally but also suprabasally in psoriasis.
NGF^40^,^51^,^52^	Over-expressed in psoriatic lesion. Stimulates keratinocyte and endothelial cell proliferation and adherence molecule expression. A marked up-regulation of NGF receptors, p75 neurotrophin receptor and tyrosine kinase A, in the terminal cutaneous nerves of psoriatic lesions. NGF and substance P may contribute to the activation of T cells.
Endothelin 1^35^–^53^	It is mitogenic to keratinocytes and a chemo-attractant to neutrophils Serum levels of endothelin-1 correlate PASI (Psoriasis Area and Severity Index) scores.
IL-23^54^–^56^	It is main inducer of the Th-17 cells and also activates nuclear STAT-3 transcription. Causes an increase in the levels of IL-17 and IL-22. Causes marked acanthosis and mixed infiltration.
IL-22^57^–^59^	Synergistically with IL-17 it induces defensins, MMPs and other molecules, including S100A7 which enhances keratinocyte mobility. IL-22 also increases mRNA expression of TNF-α.
IL-17^60^–^62^	Enhances the surface expression of the intracellular adhesion molecule-1 (ICAM-1) in fibroblast.

Recently another chemokine CX3CL1 called Fractalkine (in humans) or neurotactin (in mice) has been identified in Psoriasis. CX3CL1 is produced as a long protein (with 373-amino acid in humans) with an extended mucin-like stalk and a chemokine domain on top. The mucin-like stalk permits it to bind to the surface of certain cells. However a soluble (90 kD) version of this chemokine has also been observed. Soluble CX3CL1 potently chemo-attracts T cells and monocytes, while the cell-bound chemokine promotes strong adhesion of leukocytes to activated endothelial cells, where it is primarily expressed. Fractalkine binding to its seven-transmembrane domain G protein coupled receptor CX3CR1 triggers signalling, but it also directly mediates cell adhesion.[63,64] CX3CL1 is expressed within the brain, heart, lung, kidney, muscle and testis where it interacts with a single GPCR, CX3CR1 to trigger chemotaxis and adhesion of CX3CR1 expressing cells, including neutrophils, monocytes, NK cells and Th-1 polarized T cells.[65]

## Conclusion

For decades, the ongoing controversy on the molecular nature, choreography and hierarchy of these complex interactions e.g., between epidermal keratinocytes, T cells, neutrophils, endothelial cells and sensory nerves has served as a driving force propelling investigative dermatology to ever-new horizons. There is no question that advances in understanding the cellular immunology and biology of psoriasis, when coupled with the biotechnology revolution and rapid advances derived from human genetic studies of autoimmunity, have enhanced insights into the cause and treatment of psoriasis. The disease starts with the activation of T lymphocyte with an unknown antigen or gene product. T cell activation depends on its binding with APC (antigen presenting cell). T cells express the cell receptor known as TCR (T cell receptor), which recognizes the peptide being presented by the APC in the grove of MHC complex. The antigen stimulated activation leads to the conversion of naïve T-cells into an antigen specific cell, which may develop into a memory cell that circulate in the body [Figure 1]. After the activation of T cells, a cascade of cytokines viz. GMCSF (granulocyte macrophage colony stimulating factor), EGF, IL-1, IL-6, IL-8, IL-12, IL-17, IL-23, Fractalkine, TNF-α etc. are secreted by the activated T Cells. Due to effect of these cytokines there is keratinocyte proliferation, neutrophil migration, potentiation of Th-1 type response, angiogenesis, up-regulation of adhesion molecule and epidermal hyperplasia. We still lack a complete cure for this common and enigmatic disease, and we have not unequivocally identified genes or antigens responsible for its occurrence worldwide. Currently newer therapies are in pipeline, including an anti-IL23/22 fully humanized antibody, have also shown promise in treating psoriasis via both Th1 and Th17 pathways. But results of clinical trials will only prove its efficacy as long-term cure.
